# Copper transport system and response to ovarian cancer chemotherapy

**DOI:** 10.1186/1897-4287-13-S2-A9

**Published:** 2015-11-26

**Authors:** Karolina Tecza, Jolanta Pamula-Pilat, Zofia Kolosza, Ewa Grzybowska

**Affiliations:** 1 Center for Translational Research and Molecular Biology of Cancer, Maria Sklodowska-Curie Memorial Cancer Center and Institute of Oncology, Gliwice Branch, Poland; 2 Department of Epidemiology and Silesia Cancer Registry, Maria Sklodowska-Curie Memorial Cancer Center and Institute of Oncology, Gliwice Branch, Poland

## 

Copper is the trace element essential for the proper functioning of the cells because of its role as cofactor of many crucial enzymes, such as cytochrome c oxidase, superoxide dismutase and lysyl oxidase. Cellular transport system ensures the exact distribution of copper throughout the body and consequently its malfunction could lead to serious medical conditions, such as Menkes and Wilson disease. Apart from copper transport this system is used to move platinum and its derivates through the cell and body- including the widely used chemotherapeutic drug cisplatin. It is therefore believed that polymorphic variants in genes encoding the importer (CTR1) and intracellular exporters *via *the TNG network (ATP7A and ATP7B) could alter the drug availability and its therapeutic concentration. As the result of such alterations cisplatin resistance or oversensitivity could be developed leading to cancer therapy failure and/or serious deterioration of patients' condition. Similar consequences could also be the result of modifications in genes encoding multidrug and toxin extrusion proteins (MATE family). These efflux transporters are not the part of the main copper transport system, but are crucial for efficient elimination of toxins, including copper and platinum drugs, in liver and kidneys.

Impact of genetic polymorphisms in copper transport systems on the response to cancer treatment was analysed in the group of 129 women diagnosed with epithelial ovarian cancer receiving cisplatin-based first-line chemotherapy. For this study we selected 11 functional variants in *CTR1, ATP7A, ATP7B, MATE1 *and *MATE2-K *genes.

The results show that decrease of platinum importer *CTR1 *expression, as the consequence of intronic rs12686377 variant, leads to platinum-resistant phenotype and significantly rises the risk of death (HR 2.62; p = 0.005). On the other hand three functional variants in ATP7B transporter gene are responsible for treatment-related, both overall and early, neutropenia. Furthermore the risk of bone marrow damage increases with the accumulation of unfavourable genotypes, reaching OR 7.89 for overall and OR 27.33 for early neutropenia. Chemotherapy-induced liver damage seems to correlate with rs2289669 variant in hepatocytes guardian MATE1, which in combination with polymorphism p.Arg72Pro in *TP53 *gene is responsible for over 17-fold increase of hepatotoxicity risk.

The results indicate that efficient platinum influx to the cells is crucial for positive reaction to treatment and patients' longer overall survival. Cisplatin-induced toxicity on the other hand seems to be dependent on the management of the drug's concentration- by both intracellular transport (ATP7B) and extrusion (MATE1) systems.

## Acknowledgements

This work was financially supported by Cancer Centre and Institute of Oncology in Gliwice institutional grant- 2013 edition.

